# Dynamic FDG-PET/CT in the Initial Staging of Primary Breast Cancer: Clinicopathological Correlations

**DOI:** 10.1007/s12253-019-00641-0

**Published:** 2019-04-03

**Authors:** Kornélia Kajáry, Zsolt Lengyel, Anna-Mária Tőkés, Janina Kulka, Magdolna Dank, Tímea Tőkés

**Affiliations:** 1 Pozitron PET/CT Center, Hunyadi J. Str. 9, Budapest, H-1117 Hungary; 2 Semmelweis University 2nd Department of Pathology, Üllői str. 93., Budapest, H-1091 Hungary; 3 Semmelweis University Oncology Center, Tömő utca 25-29, 4th floor, Budapest, H-1083 Hungary

**Keywords:** Breast cancer, Kinetics, PET-CT, Tumor-infiltrating lymphocytes

## Abstract

Our aim was to evaluate correlation between clinicopathological features (clinical T and clinical N stages; histological type; nuclear grade; hormone-receptor and HER2 status, proliferation activity and tumor subtypes) of breast cancer and kinetic parameters measured by staging dynamic FDG-PET/CT examinations. Following ethical approval and patients’ informed consent we included 34 patients with 35 primary breast cancers in our prospective study. We performed dynamic PET imaging, and assessed plasma activity noninvasively. To delineate primary tumors we applied a frame-by-frame semi-automatic software-based correction of motion artefacts. FDG two-compartment kinetic modelling was applied to assess K1, k2, k3 rate coefficients and to calculate Ki (tracer flux constant) and MRFDG (FDG metabolic rate). We found that k3, Ki and MRFDG were significantly higher in higher grade (*p* = 0.0246, 0.0089 and 0.0076, respectively), progesterone-receptor negative (*p* = 0.0344, 0.0217 and 0.0132) and highly-proliferating (*p* = 0.0414, 0.0193 and 0.0271) tumors as well as in triple-negative and hormone-receptor negative/HER2-positive subtypes (*p* = 0.0310, 0.0280 and 0.0186). Ki and MRFDG were significantly higher in estrogen-receptor negative tumors (*p* = 0.0300 and 0.0247, respectively). Ki was significantly higher in node-positive than in node-negative disease (*p* = 0.0315). None of the assessed FDG-kinetic parameters showed significant correlation with stromal TIL. In conclusion, we confirmed a significant relationship between kinetic parameters measured by dynamic PET and the routinely assessed clinicopathological factors of breast cancer: high-grade, hormone-receptor negative tumors with high proliferation rate are characterized by higher cellular FDG-uptake and FDG-phosphorylation rate. Furthermore, we found that kinetic parameters based on the dynamic examinations are probably not influenced by stromal TIL infiltration.

## Introduction

In locally advanced breast cancer current guidelines suggest 2-deoxy-2[18F]fluoro-D-glucose positron emission tomography and computer tomography (FDG-PET/CT) as a useful and accurate tool for staging. Additionally, its role is also emerging in the response evaluation during neoadjuvant therapies [1]. Most commonly PET/CT scans are evaluated semi-quantitatively: the amount of the accumulated radiotracer is measured at one predefined time-point after injection (i.e. static imaging) and characterized by the calculated Standardized Uptake Value (SUV). SUV shows strong correlation with the clinicopathological characteristics of breast cancer, such as histological tumor type, biological subtype, grading and proliferation rate [2, 3]. Nonetheless, the static nature of SUV measurement can introduce several sources of bias, most importantly it is highly dependent on the time elapsed from tracer injection as well as body composition and glucose-metabolism of the patient [4]. These limitations of the SUV parameter could cause several bias during the staging and response evaluation. Therefore, in our current study we used dynamic PET imaging to accurately quantify the radiotracer uptake by the kinetic analysis of FDG-accumulation over a period of time.

In case of dynamic breast PET imaging, data collection lasted constantly for 60 min, right directly after the tracer injection. The compartment modelling of FDG-distribution is used to evaluate the data acquired by dynamic imaging. Using the FDG-two-compartment model we could estimate the important rate parameters of the tracer flow between the blood-pool (first, *plasma compartment*) and the investigated tissue compartments (i.e. a *transfer compartment*, where FDG is intracellular and a *metabolic compartment* where FDG is in FDG-6-phosphate form). The estimated rate constants describe the influx and efflux of the FDG between these compartments: K1 and k2 are the rate constants representing carrier-mediated transport of FDG from plasma to tissue (K1) and back from tissue to plasma (k2), in the transfer compartment. k3 represents the rate constant for phosphorylation of the FDG by the hexokinase enzyme in the metabolic compartment. Based on the above listed parameters the net tracer influx constant [Ki = (K1*k3) / (k2 + k3)] could be calculated. [5, 6].

We aimed to investigate the possible role of dynamic imaging in the characterization of breast cancer during the initial clinical staging. We assessed the correlations between the kinetic parameters measured by dynamic PET examinations (i.e. K1, k2, k3, Ki) and the routinely used predictive and prognostic factors of breast cancers − such as clinical TNM, histological tumor type, tumor grade, proliferation rate and receptor status (i.e. hormone-receptor (HR) and human epidermal growth factor receptor 2 (HER2) expression). We also defined the clinical subtypes of the tumors, due to their important role in therapeutic choices in the daily practice [7]. Moreover, a novel pathological marker was also assessed in our study: the presence of tumor infiltrating lymphocytes (TIL) in the stroma of the tumors. Immune-cells are playing a central role in shaping of tumor microenvironment and presence of TILs proved to be a possible prognostic factor for favorable clinical outcome by itself [8]. Additionally, the lymphocyte predominant subgroups of triple negative tumors and hormone-receptor negative and HER2-positive (HR-/HER2+) tumors show a favorable response to systemic therapy, but, interestingly, this is not the case in hormone-receptor positive breast cancers. TIL could also have a future role to predict the effectiveness of immunotherapies [8]. In our study we tried to investigate if there is a connection between the presence of TILs and the measured FDG-influx during dynamic PET examinations.

In summary, in our current paper we present the results of our study with dynamic FDG-PET/CT imaging used in the staging of breast cancer. Our focus is on – over the routinely assessed clinicopathological characteristics of breast tumors – the rarely investigated correlations between tumor phenotype and FDG-kinetics, with special emphasis on clinical subtypes and tumor microenvironment (characterized by stromal TILs). We also plan to extend our research to the response evaluation during neoadjuvant therapies.

## Patients and Methods

### Patient Enrollment

In our prospective study we included patients with invasive breast cancer. The inclusion criteria were the same as in our earlier published study [9], as follows: 1) diagnosis of primary breast cancer confirmed by core biopsy; (2) patients who were referred to the multidisciplinary tumor board of Semmelweis University Oncology Center in whom the board acknowledged the need of whole-body staging by using FDG-PET/CT; (3) only tumors of at least 20 mm (T1c≤) were included to avoid the negative impact of partial volume effect in case of smaller lesions [10]; (4) patients with ECOG performance status 0 or 1 and age above 18 years; (5) signed written informed consent form.

Patients were excluded from the current analysis if: (1) any distant metastasis of parenchymal tissues was confirmed by staging FDG-PET/CT examinations (the presence of bone metastases was permitted); (2) chemotherapy was already initiated or radiation therapy was applied previously in the region of the affected breast; (3) inflammatory breast cancer; (4) pregnant or breast feeding patients; (5) claustrophobia; (6) patients who were not able or did not want to lie still for 60 min during the examination – especially with known co-morbidities which interfere with the need of prolonged immobility (Parkinson disease, musculoskeletal disorders etc.); (7) if a peripheral venous access could not be ensured. Only those patients can be considered for dynamic PET who are capable of successful cooperation during the whole, prolonged time of the imaging procedure.

Clinical TNM (cTNM stages) was assessed by using routinely applied diagnostic imaging modalities (X-ray and ultrasound mammography, and FDG-PET/CT) according to American Joint Committee on Cancer (AJCC) TNM 8th guideline [11].

Ethical approval for the study was given by the Semmelweis University Institutional Review Board (SE-TUKEB No.119/2013; date of approval: 24 June 2013). Every patient was informed personally and written informed consent form was signed by them before the enrollment.

### Histopathological Analysis

Formalin-fixed, paraffin-embedded pre-treatment core biopsy samples were examined at the Semmelweis University 2nd Department of Pathology, by board-certified pathologists. Detailed histological characterization was performed on the core biopsy samples, including the determination of histological type and nuclear grade. Immunohistochemistry (IHC) was routinely performed to evaluate hormone receptor – estrogen (ER) and progesterone (PR) – status, as well as HER2 expression according to international guidelines, with the evaluations of tumor proliferation by using the Ki-67 labeling index (Ki-67 LI).

Hormone receptor positivity was confirmed if Allred score was above or equal to 3 [12]. HER2 overexpression was defined as IHC 3+. HER2 1+ or 0 tumors were considered HER2 negative. For IHC 2+ samples, fluorescent in situ hybridization (FISH) was performed to confirm gene amplification. HER2 status was defined according to the American Society of Clinical Oncology/College of American Pathologists (ASCO/CAP) Guideline valid at the time of diagnosis, i.e. HER2-positive patients treated between January 2008 and November 2013 were identified according to the 2007 ASCO/CAP Guideline [13] and from then on according to the Guideline published in October 2013 [14]. Ki-67 LI was scored as described by Dowsett et al. [15, 16]: every slide was assessed visually and the proportion of positive cells was determined by counting approximately 500 tumor cells at 400x magnification. For Ki-67 LI a cell was considered positive if any nuclear signal was observed, similar to the earlier published studies of Tőkés et al. [17].

Biological subtype of the tumors was defined according to the recommendations of the 2017 St. Gallen International Breast Cancer Conference [7]. Triple negative (TNBC), hormone-receptor negative and HER2-positive (HR-/HER2+), hormone-receptor positive and HER2-positive (HR+/HER2+) and hormone-receptor positive and HER2-negative (HR+/HER2-) tumors were separated based on the expression of ER, PR and HER2, taking into account the Ki-67 LI status, as well.

Additionally, the core-biopsy samples were also evaluated for the presence of tumor infiltrating lymphocytes (TIL) in the stroma of the tumors, according to the TILs Working Group guideline published in 2015 [8]. Evaluation of the stromal TILs was performed by co-authors TAM and JK, experts of the field.

### PET/CT Studies

All PET examinations were performed with Siemens Biograph TruePoint 6 HD (Siemens Molecular Imaging Division, Knoxville, USA) PET/CT scanner. Patients were prepared according to standard guidelines [18, 19]. Most importantly, the examinations were preceded by at least 6-hour starvation. The level of blood glucose and body-weight were measured upon arrival to the PET/CT Center. The average weight of the investigated patients (*n* = 34) was 67.7 ± 12.5 kg and the mean blood glucose was 5.1 ± 0.9 mmol/L. The scans were acquired after the intravenous injection of 155.4–384.8 MBq (4.2–10.4 mCi) FDG according to body weight (~3.7 MBq/kg; ~0.1 mCi/kg). The mean dose of administered FDG was 254.6 ± 51.9 MBq (6.8 ± 1.4 MBq). Intravenous injection of tracer was performed with the patient lying on the examination table (minute zero). PET data collection was continuous (list mode) from minute zero, with fixed bed position over the chest region (Z axis length: 21.6 cm). Dynamic PET data acquisition lasted for 60 min, with the patient lying still on the examination table of the PET/CT scanner. The dynamic imaging sequence, starting with the initiation of the bolus was 8 × 15 seconds, 8 × 30 seconds, 2 × 60 seconds, 3 × 2 minutes, 4 × 3 minutes, 1 × 4 minutes and 6 × 5 minutes. PET images were corrected for decay, attenuation and normalization, scatter and randoms rejection, as well as for dead time. PET data were acquired in three-dimensional mode and were reconstructed using CT data for attenuation correction with ordered subsets expectation maximization (OSEM) algorithm (4 iterations, 8 subsets), using a 168x168x74 image matrix with voxel dimension of 4.07 × 4.07 × 3.0 mm. Image reconstruction was performed as described earlier in our previous study [9]: the early frames of every dynamic study (from 1 to 4, from 5 to 8, from 9 to 10 and from 11 to 12 frames, respectively) was averaged in a time-weighted manner (which process is offered by most of the available software tools) to correct the earlier described inaccuracies without significant degradation of time resolution.

Collected dynamic data were analyzed by using PMOD software (v3.310, Zürich, Switzerland). Every study was assessed by the same two experienced observers (KK and TT). To generate the time-activity-curves (TACs), changes of activity concentrations were assessed in the tumor and in the blood pool (i.e. plasma). To assess plasma activity, observers draw a sphere volume of interest (VOI) in the left ventricle (LV) of the heart sized 15 mm in diameter. Similarly, 15 mm sized sphere VOIs were also used to measure the primary tumors’ activity over time. To eliminate movement artefacts due to involuntary movements and breathing, positions of the VOIs were corrected by using a semi-automatic, computer-assisted method, published earlier [9]: at first we placed a VOI over the tumor, which exceeded the longest diameter of the examined lesion with at least 10 mm, and then we centered the VOI to the voxel with the highest uptake in the target region. By reducing the size of the VOI to 15 mm in diameter, we finalized the motion correction of the current time-frame. This procedure was repeated on every time-frame of the dynamic studies. This approach seemed to be reliable, reproducible, with low inter-observer variability, compared to manual motion correction [9].

Thereafter TACs were defined by using the VOIs of left ventricle and the tumor(s), afterwards FDG two-compartment kinetic modelling (non-reversible, k4 = 0) was applied to assess K1, k2, k3 coefficients and to calculate Ki and MRFDG (the FDG metabolic rate in the tissue, μmol/min/100 g tissue). Relationship of these parameters is defined by the following equation:

$$ \mathrm{MRFDG}={\mathrm{C}}_{\mathrm{glc}}\mathrm{x}\ {\mathrm{K}}_{\mathrm{i}} $$

where C_glc_ is the blood glucose level of the patient measured right before the initiation of the dynamic PET imaging. It should be highlighted that MRFDG is not equivalent to the glucose metabolic rate (referred as MRGlu), since the constant which describes the relationship between the FDG accumulation and glucose consumption (i.e. the lumped constant) is not defined yet in case of breast tissues - therefore the exact rate of the glucose metabolism cannot be calculated reliably [5, 20].

### Statistical Analysis

Data were expressed as mean ± standard deviation (SD) or median and lower interquartile-upper interquartile (LIQ-UIQ). All applied statistical tests were two-sided and *p* values<0.05 were considered significant. Shapiro-Wilks test was applied to test the normality of the data. Connections between clinicopathological characteristics and the kinetic parameters were assessed by using Mann-Whitney and Kruskal-Wallis tests.

For data collection and processing we used Microsoft Excel 2010 (Microsoft Corporation, Redmond Washington, USA), Statistica v13.2 (StatSoft Inc.) and SPSS v22.0 (IBM Corporation) software.

## Results

A total of 35 lesions detected during 34 dynamic studies were included in this current analysis. Dominantly grade 3 (19/35; 54%) invasive breast carcinomas of no special type (IBC NST) (30/35; 85.7%) were evaluated. 24 of the included tumors were ER positive (69%) and 22 were PR positive (63%) (total number of hormone receptor positive (HR+) lesions was 25 – one lesion showed solely PR positivity, but ER negativity). Only six tumors showed HER2 positivity (17%) and 27 tumors showed elevated proliferation activity, i.e. Ki-67 LI > 20% (77%). (Table 1).

**Table 1:** Clinicopathological characteristics (*n* = 34 patients with *n* = 35 lesions)

Clinical TNM (*n* = 34 patients)	No.	%
Clinical T stage		
cT1c	4	11.8
cT2	25	73.5
cT3	5	14.7
Clinical N stage		
cN0	9	26.5
cN positivity (cN1–3)	25	73.5
Clinical M stage		
cM0	33	97
cM1 *	1	3
Pathological Characteristics (*n* = 35 lesions)	No.	%
Histology		
IBC-NST	30	85.7
Invasive lobular carcinoma	2	5.7
Other †	3	8.6
Grade ‡		
1	2	5.7
2	8	22.9
3	19	54.3
Estrogen receptor status		
ER positive	24	68.6
ER negative	11	31.4
Progesterone receptor status		
PR positive	22	62.9
PR negative	13	37.1
HER2 status		
HER2 positive	6	17.1
HER2 negative	29	82.9
Proliferation rate		
High Ki-67 LI	27	77.1
Low Ki-67 LI	8	22.9
Clinical subtypes*#*		
Triple negative (TNBC)	8	22.9
HR-/HER2+	2	5.7
HR+/HER2+	4	11.4
HR+/HER2-: Luminal A-like tumors	5	14.3
HR+/HER2-: Intermedier subgroup	9	20
HR+/HER2-: Luminal B-like tumors	7	25.7

*1 patient was included with bone metastases on the initial staging

†two invasive pleiomorph carcinoma, one undifferentiated adenocarcinoma of the breast

‡in 6 patients tumor grade is unknown . # 1 patient has a PR-positive but ER-negative tumor, therefore the overall number of HR+ lesions is 25

*Abbreviations*: *IBC NST* invasive breast carcinomas of no special type, *ER* estrogen receptor, *PR* progesterone receptor, *HER2* human epidermal growth factor receptor 2, *Ki-67 LI* Ki-67 labeling index, *HR-/HER2+* hormone-receptor negative and HER2-positive, *HR+/HER2+* hormone-receptor positive and HER2-positive, *HR+/Her2-* hormone-receptor positive and HER2-negative

Regarding clinical subtypes of the investigated lesions, eight tumors were TNBC (22.9%) and 2 were HR-/HER2+ (5.7%). 4 tumors were HR+/HER2+ (11.4%). The remaining 21 lesions were HR+/HER2-. Amongst these, five were luminal A-like tumor (14.3%), seven were luminal B-like (20%) and 9 lesions belonged to the intermediate subgroup (25.7%).

We analyzed the relationship between the kinetic parameters measured on the dynamic PET/CTs and the known predictive and prognostic factors (i.e. clinical T and clinical N stages; histological type; nuclear grade; ER, PR and HER2 status, Ki-67 LI and tumor subtype) in pre-therapy core-biopsies (Table 2).

**Table 2:** Relationship between the clinicopathological characteristics of the investigated breast tumors and the kinetic parameters of the performed dynamic PET examinations

Feature (No. of tumors)	K1 mean	k2 mean	k3 mean	Ki mean	MRFDG mean
Clinical T stage					
T1c (4)	0.185	0.543	0.062	0.018	8.701
T2 (26)	0.375	1.095	0.099	0.036	17.531
T3 (5)	0.258	0.779	0.164	0.044	23.292
*p* value	**0.3742**	**0**.**9003**	**0.2206**	**0.0834**	**0.0780**
Clinical N stage					
N0 (9)	0.292	0.909	0.076	0.023	12.961
N1–3 (26)	0.352	1.014	0.114	0.039	18.862
*p* value	**0.5459**	**0.2271**	**0.2575**	**0.0315**	**0.1046**
Histology *					
IBC-NST (30)	0.337	1.004	0.101	0.034	16.951
other (5)	0.333	0.884	0.125	0.041	19.709
Nuclear grade †					
1 (2)	0.149	0.427	0.049	0.015	6.148
2 (8)	0.353	0.983	0.071	0.022	10.838
3 (19)	0.371	1.157	0.129	0.042	21.875
*p* value	**0.3075**	**0.3417**	**0**.**0246**	**0.0089**	**0.0076**
ER status					
positive (24)	0.279	0.727	0.094	0.032	14.934
negative (11)	0.462	1.555	0.128	0.041	22.604
*p* value	**0.4095**	**0.5872**	**0.0667**	**0.0300**	**0.0247**
PR status					
positive (22)	0.277	0.735	0.088	0.029	13.775
negative (13)	0.436	1.414	0.132	0.044	23.386
*p* value	**0.2035**	**0.5782**	**0.0344**	**0.0217**	**0.0132**
HER2 status					
positive (6)	0.369	1.136	0.104	0.033	15.824
negative (29)	0.329	0.956	0.105	0.035	17.659
*p* value	**0.5933**	**0.2184**	**0.4801**	**0.8814**	**0.9151**
Ki-67 LI					
low (8)	0.269	0.802	0.062	0.021	9.931
high (27)	0.356	1.042	0.117	0.039	19.542
*p* value	**0.8323**	**0.9843**	**0.0414**	**0.0193**	**0.0271**
Subtype					
Triple negative (8)	0.456	1.602	0.134	0.043	23.844
HR-/HER2+ (2)	0.674	2.087	0.136	0.041	21.863
HR+/HER2+ (4)	0.217	0.661	0.088	0.029	12.804
HR+/HER2- (21)	0.281	0.710	0.093	0.032	15.303
*p* value (all)	**0.1932**	**0.2396**	**0.1666**	**0.1865**	**0.1374**
*p* value (TNBC and HER2+ vs. all HR+)	**0.1514**	**0.2703**	**0.0310**	**0.0280**	**0.0186**
TIL‡					
TIL ≤1% (8)	0.225	0.483	0.089	0.033	15.346
1% < TIL <20% (12)	0.347	1.023	0.109	0.036	18.807
TIL ≥20% (3)	0.742	2.916	0.095	0.032	16.048
*p* value (all)	**0.4713**	**0.0940**	**0.2055**	**0.5849**	**0.4346**

*p* values: in bold. Underlined: statistically significant difference

*Due to low case numbers and statistical power we did not perform a statistical comparison;

† Missing in case of 6 tumors; ‡ missing in case of 12 tumors

*Abbreviations*: *IBC NST* invasive breast carcinomas of no special type, *ER* estrogen receptor, *PR* progesterone receptor, *HER2* human epidermal growth factor receptor 2, *Ki-67 LI* Ki-67 labeling index, *TNBC* triple negative, *HR−/ HER2+* hormone-receptor negative and HER2-positive, *HR+/ HER2+* hormone-receptor positive and HER2-positive, *HR+/ HER2+* hormone-receptor positive and HER2-negative, *TIL* tumor infiltrating lymphocytes

We did not find significant differences according to tumor size (clinical T categories). However, in case of nodal involvement Ki was significantly higher in node positive than in node negative disease (*p* = 0.0315).

Tumors with higher nuclear grade showed significantly higher k3 (*p* = 0.0246), Ki (*p* = 0.0089) and MRFDG (*p* = 0.0076).

Regarding hormone receptor status, we found that Ki (*p* = 0.0300 and *p* = 0.0217, respectively) and MRFDG (*p* = 0.0247 and *p* = 0.0132, respectively) were significantly higher in ER negative and PR negative tumors than in hormone receptor positive (HR+) cancers. In PR negative tumors, k3 was also significantly higher compared to PR positive lesions (*p* = 0.0344). HER2 status did not show any correlation with the kinetic parameters. Tumors with elevated Ki-67 LI (i.e. high proliferation activity) showed significantly higher k3 (*p* = 0.0414), Ki (*p* = 0.0193) and MRFDG (*p* = 0.0271).

Additionally, FDG-kinetics were also compared between the St. Gallen 2017 consensus [7] based clinical subtypes. Initially, we did not find significant differences between the clinical subtype distribution of the tumors and the investigated kinetic parameters. However, considering the low number of cases present in each clinical subtype, we performed an additional analysis: subgroups were compared based on their prognostic significance - more aggressive TNBC and HR-/HER2+ subtypes (*n* = 10) were compared with hormone-receptor positive lesions (HR+/HER2+ and HR+/HER2- tumors, *n* = 25). TNBC and HR-/HER2+ subtypes showed significantly higher k3 (*p* = 0.0310), Ki (*p* = 0.0280) and MRFDG (*p* = 0.0186) compared to hormone-receptor positive diseases.

**Fig. 1:**
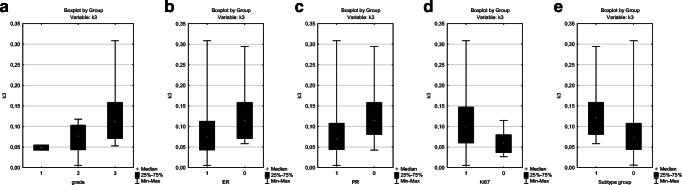
Box-plot figures representing the comparison of k3 values between (**a**) grade 1, grade 2 and grade 3 tumors (*p*=0.0246); (**b)** estrogen (ER) positive(+) and ER negative(−) tumors (*p*=0.0667); (**c**) progesterone (PR) positive(+) and PR negative(−) tumors (*p*=0.0344); (**d**) tumors with high and low Ki-67 LI (*p*=0.0414). We also compared the value of k3 between two patient groups based on biological subtypes (**e**): the more aggressive TNBC and HR-/HER2+ subtypes(group 1) and the hormone-receptor positive lesions (HR+/HER2+ and HR+/HER2- tumors) (group 2) (*p*=0.0310)

**Fig. 2:**
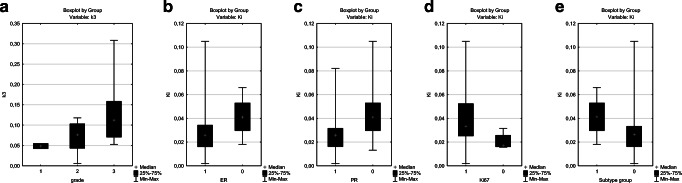
Box-plot figures representing the comparison of Ki values between (**a**) grade 1, grade 2 and grade 3 tumors (*p*=0.0089); (**b**) estrogen (ER) positive(+) and ER negative(−) tumors (*p*=0.0300); (**c**) progesterone (PR) positive(+) and PR negative(−) tumors (*p*=0.0217); (**d**) tumors with high and low Ki-67 LI (*p*=0.0193). We also compared the value of Ki between two patient groups based on biological subtypes (**e**): the more aggressive TNBC and HR-/HER2+ subtypes(group 1) and the hormone-receptor positive lesions (HR+/HER2+ and HR+/HER2- tumors) (group 2) (*p*=0.0028)

**Fig. 3:**
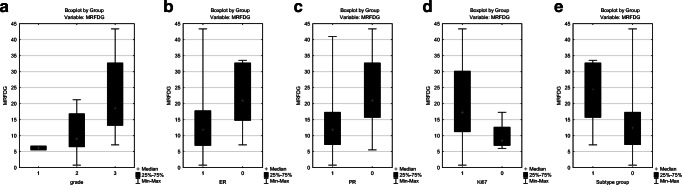
Box-plot figures representing the comparison of MRFDG values between (**a**) grade 1, grade 2 and grade 3 tumors (*p*=0.0076); (**b**) estrogen (ER) positive(+) and ER negative(−) tumors (*p*=0.0247); (**c**) progesterone (PR) positive(+) and PR negative(−) tumors (*p*=0.0132); (**d**) tumors with high and low Ki-67 LI (p=0.0271). We also compared the value of MRFDG between two patient groups based on biological subtypes (**e**): the more aggressive TNBC and HR-/HER2+ subtypes(group 1) and the hormone-receptor positive lesions (HR+/HER2+ and HR+/HER2- tumors) (group 2) (*p*=0.0186)

In summary, three kinetic parameters (k3, Ki and MRFDG) were strongly associated with the biological behavior of the tumors. The correlations between k3, Ki and MRFDG and tumor grade, ER and PR status, Ki-67 LI and subtype groups are presented on Figs. 1, 2, and 3. All of these correlations were significant except the correlation between k3 and ER status.

We also analyzed the correlations between the presence of stromal TILs and kinetic parameters of the primary tumors. Data about the presence TIL was available in 23 patients. Median TIL was 5% (LIQ-UIQ:1–7.5%), most of the lesions showed slightly elevated but not significantly high stromal TIL content (12/23 lesions showed TIL between 1% and 20%). Every investigated kinetic parameter was higher in tumors with TIL infiltration >1% and higher K1 and k2 was measured in tumors with increased TIL (>20%), but none of these observations were statistically significant (Table 2).

## Discussion

In breast cancer, the routinely used predictive and prognostic (bio)markers are mainly based on invasive tumor sampling and pathological assessment (i.e. tumor type, grade, proliferation activity, receptor status etc.). The relationship between these markers and FDG-avidity measured during static FDG-PET/CT imaging is already known and substantially investigated [2, 3]. Amongst others, our research group already highlighted the strong relationship between SUV and several clinicopathological factors of breast cancer: lesions with IBC-NST phenotype showing high-grade features and increased proliferation, with triple negativity and axillary lymph node positivity usually show higher FDG-uptake compared to low-grade, luminal A-like tumors with node negativity [2, 3].

On the contrary, the role of dynamic PET imaging is under debate in breast cancer. The indisputable advantage of dynamic FDG-PET/CT is its capability to accurately quantify FDG-uptake. Therefore, dynamic imaging carries a potential to define novel predictive and prognostic biomarkers in vivo for numerous tumor types. The assessment of the correlations between in vivo measured parameters of FDG tracer kinetics and biological behavior of breast tumors can increase the value of dynamic PET/CT imaging in the daily practice. However, implementation of dynamic PET imaging is complicated, time- and resource-consuming; therefore, the delineation of a suitable patient population is essential. A few studies already investigated the role of the parameters derived from dynamic data collection followed by tracer kinetic modelling in the staging of breast cancer [21] as well as during response evaluation to neoadjuvant therapy [20–27]. Our study focused on the rarely investigated correlations between tumor phenotype and FDG-kinetics, with special emphasis on clinical subtypes and tumor microenvironment (characterized by stromal TILs), which has gained special attention in recent years of cancer research.

In our study, while analyzing core-biopsy samples of breast cancer, we did not find any correlation between lesion size and the kinetic parameters of the dynamic PET studies. If axillary lymph node status was positive per clinical-imaging investigations (>cN0), every measured kinetic parameter was higher compared to clinically node-negative (cN0) cases, but the relationship was only significant in case of Ki. Dunnwald et al. described similar results in their earlier published study regarding Ki, but they found that K1 is also significantly higher in node positive disease (nonetheless, they did not published any results particularly about the correlations with k2 and k3) [21].

Regarding the biological characteristics of the assessed breast cancers, we found that every assessed kinetic parameter was elevated in high-grade tumors and this correlation was statistically significant in case of k3, Ki and MRFDG. When assessing the correlation with tumor proliferation, similar correlations were found: lesions with high Ki-67 LI showed significantly higher values of k3, Ki and MRFDG compared to lesions characterized by low Ki-67 LI. Dunnwald et al. found the same significant correlations for Ki. They also assessed the relationship between Ki-67 LI and K1, but did not find a significant correlation with tumor grade or proliferation (it is note that they did not evaluate the role of k2 nor k3) [21].

Regarding hormone receptor status, significantly different FDG-kinetic parameters were detected in HR- tumors compared to HR+ lesions. In case of ER expression, Ki and MRFDG were significantly higher in ER-negative than in ER-positive lesions. Regarding PR the k3, Ki and MRFDG was significantly higher in PR-negative than in PR-positive tumors. In our study, we did not find a significant correlation between HER2 positivity and the investigated kinetic parameters of the dynamic studies. Nonetheless, the group of TNBC and HR-/HER2+ subtypes showed significantly higher values of k3, Ki and MRFDG in comparison to hormone-receptor positive breast cancers (i.e. HR+/HER2+ and HR+/HER2- tumors). Regarding hormone-receptor and HER2-status similar correlations were published earlier by Dunnwald et al. as was observed in our study, but only in case of Ki [21]. The analysis of the relationship between breast cancer subtypes and FDG-kinetics was not performed earlier, only in case of static PET/CT imaging, where similar correlations were described [2, 3]. In our study TNBC and HR-/HER2+ subtypes - which are characterized by worse clinical outcome compared to hormone-receptor positive (HR+) tumors – are confirmed to have high FDG-influx and a more prominent FDG metabolic rate, which explains the significantly higher SUVs of these phenotypes during routine static PET imaging [2, 3].

We also assessed the ratio of stromal TILs in the primary tumors, due to its possible prognostic and predictive role in breast cancer [8]. It is reasonable to hypothesize that tumor microenvironment - especially the amount of stromal TILs - could affect the FDG uptake and distribution of malignant lesions. In gastric cancer [28] and in case of non-small cell lung cancer [29] a positive correlation was already described between the presence of TILs and FDG-uptake in case of routinely used static FDG-PET/CT imaging. Regarding breast cancer, this question was only assessed indirectly, in the recently published study of Fujii et al. [30], who found a relationship between higher neutrophil granulocyte/lymphocyte ratio (i.e. a marker of systemic inflammation) and higher-FDG uptake (also evaluated during static imaging). Nevertheless, in our study we did not find significant difference in the kinetic parameters of the dynamic studies when compared breast tumors with different ratios of stromal TILs. Our preliminary results suggest that the presence of stromal TILs did not affect significantly the measured FDG-kinetics of the dynamic studies in breast cancer patients – however this hypothesis must be further investigated by repeating our study in a larger group of patients.

Based on the above, we can highlight that the same correlations can be found between the kinetic parameters of the dynamic PET examinations and breast tumors’ phenotype as were described earlier when only static FDG-PET/CT imaging was used in the staging of breast cancers [2, 3]. During dynamic imaging high-grade, hormone-receptor negative tumors with high proliferation rate are characterized by higher cellular FDG-uptake and FDG-phosphorylation rate (in harmony with earlier published results [21]). These lesions are showing significantly higher SUVs if static FDG-imaging is applied for staging [2, 3]. Nevertheless, these tumors frequently present as locally advanced diseases with aggressive clinical behavior, therefore are regularly treated with neoadjuvant therapy. In this case, the limitations of the SUV parameter of static imaging could cause several bias during the response evaluation. For example if the patient lost significant body-weight during the therapeutic course or had altered renal clearance due to the applied chemotherapies the value of the SUV could be diverged accordingly [4]. Dynamic imaging however is capable to accurately quantify the FDG-uptake and it is not affected by these bias. For this reason, testing of dynamic PET has already been begun in this specific indication [20–27]. Based on these earlier studies we can conclude that an initially high rate of FDG-influx and lower MRFDG as well as a lower post-therapeutic Ki and K1 are both predictive to favorable tumor response to the neoadjuvant therapy [21–25]. These result are probably related to the strong correlations between the value of Ki and tumor vascularization [23], blood flow [24] and functional tumor volume [26], which parameters are suitable to delineate residual disease, if still onset after the neoadjuvant treatment of breast tumors. Moreover, it seems likely that response evaluation with the kinetic parameters of the dynamic studies is not depending on the initial pre-therapy FDG-uptake as much as the static evaluations (using the SUV) is. With dynamic imaging the follow-up of less FDG-avid breast tumors, with lower pre-therapy FDG-uptake can also be implemented [21, 22, 27].

Thus, based on our results, in the staging of breast cancer dynamic imaging may not have an outstanding added value due to its similar correlations with the clinico-pathological characteristics of the tumors which has already been described by static imaging studies. However, our current results are suggesting that if tumor microenvironment is taken into account, it seems likely that the parameters of dynamic imaging are not affected by TIL ratio as much as the routinely applied static imaging. Additionally, based on earlier preclinical and clinical studies, if neoadjuvant chemotherapy is planned in locally advanced disease, dynamic imaging could be a promising tool for response evaluation, by quantifying FDG-uptake more precisely when initial staging and post-therapy imaging is compared. Our next studies are addressed to further enlighten its role in the clinical routine.

The main limitations of our study is our VOI delineation method: the reported kinetic parameters only describing a 15 mm sized portion of the investigated tumors instead of the whole, sometimes heterogeneous tumor mass. Additionally, during motion correction the VOIs were centered on the most metabolically active part of the lesions, which could also be misleading, especially in large lesions. Additionally, the small number of tumors in which TIL was determined (only in 65% of the included patients) is also limiting the value of our results. To further clarify the correlation between tumor microenvironment and FDG-kinetics, our study must be repeated in larger group of patients.

In conclusion, our preliminary findings proved a significant relationship between kinetic parameters measured by dynamic PET and the routinely assessed clinicopathological factors, similarly as was known in case of static SUV measurements. We also confirmed that high-grade, hormone-receptor negative tumors with high proliferation rate are characterized by higher cellular FDG uptake and FDG phosphorylation rate based on dynamic imaging. Additionally, we further underlined the aggressive biological behavior of TNBC and HR-/HER2+ subtypes. Furthermore, we described that kinetic parameters measured by dynamic PET examinations are probably not influenced by stromal TIL infiltration.

## Data Availability

The datasets generated during and/or analysed during the current study are available from the corresponding author on reasonable reques.

## Abbreviations

AJCC: American Joint Committee on Cancer
ASCO/CAP: American Society of Clinical Oncology/College of American Pathologists
C_glc_: Blood glucose level of the patient measured right before the initiation of the dynamic imaging
ER: Oestrogen receptor
FDG-PET/CT: 2-deoxy-2[18F]fluoro-D-glucose positron emission tomography and computer tomography
FISH: Fluorescent in situ hybridization
HER2: Human epidermal growth factor receptor 2
HR: Hormone receptor (both estrogen and progesterone receptors)
HR−/ HER2+: Hormone-receptor negative and HER2-positive;
HR+/ HER2+: Hormone-receptor positive and HER2-positive;
HR+/ HER2+: Hormone-receptor positive and HER2-negative.
IBC NST: Invasive breast carcinomas of no special type
IHC: Immunohistochemistry
Ki: Net tracer influx constant
Ki-67 LI: Ki-67 labelling index
LIQ-UIQ: Lower interquartile-upper interquartile
LV: Left ventricle
MRFDG: FDG metabolic rate in the tissue, μmol/min/100 g tissue
OSEM: Ordered subsets expectation maximization
PR: Progesterone receptor
SD: Standard deviation
SE-TUKEB: Semmelweis University Institutional Review Board
SUV: Standardized Uptake Value
TAC: Time-activity-curve
TIL: Tumor infiltrating lymphocytes
TNBC: Triple negative breast cancer
VOI: Volume of interest
